# Altered miRNA cargo of endometrial extracellular vesicles in patients with endometriosis: potential implications for pregnancy outcomes

**DOI:** 10.1093/hropen/hoag040

**Published:** 2026-05-07

**Authors:** Alba Bas-Rivas, Adrián Villalba, Aitana Merino-Pérez, Amparo Faus, Irene Juárez, Carmen Vidal, Pilar Alamá, Antonio Pellicer, Ana Corachán, Irene Cervelló, Hortensia Ferrero

**Affiliations:** Fetal and Reproductive Medicine Department, Instituto de Investigación Sanitaria La Fe (IIS La Fe)/IVI-RMA Global Research Alliance, IVI Foundation, Valencia, Spain; Fetal and Reproductive Medicine Department, Instituto de Investigación Sanitaria La Fe (IIS La Fe)/IVI-RMA Global Research Alliance, IVI Foundation, Valencia, Spain; Fetal and Reproductive Medicine Department, Instituto de Investigación Sanitaria La Fe (IIS La Fe)/IVI-RMA Global Research Alliance, IVI Foundation, Valencia, Spain; Fetal and Reproductive Medicine Department, Instituto de Investigación Sanitaria La Fe (IIS La Fe)/IVI-RMA Global Research Alliance, IVI Foundation, Valencia, Spain; Department of Gynaecology, Hospital Universitari i Politècnic La Fe, Valencia, Spain; Fetal and Reproductive Medicine Department, Instituto de Investigación Sanitaria La Fe (IIS La Fe)/IVI-RMA Global Research Alliance, IVI Foundation, Valencia, Spain; Reproductive Medicine Department, IVI-RMA, Valencia, Spain; Fetal and Reproductive Medicine Department, Instituto de Investigación Sanitaria La Fe (IIS La Fe)/IVI-RMA Global Research Alliance, IVI Foundation, Valencia, Spain; Reproductive Medicine Department, IVI-RMA, Valencia, Spain; Reproductive Medicine Department, IVI-RMA, Rome, Italy; Fetal and Reproductive Medicine Department, Instituto de Investigación Sanitaria La Fe (IIS La Fe)/IVI-RMA Global Research Alliance, IVI Foundation, Valencia, Spain; Fetal and Reproductive Medicine Department, Instituto de Investigación Sanitaria La Fe (IIS La Fe)/IVI-RMA Global Research Alliance, IVI Foundation, Valencia, Spain; Fetal and Reproductive Medicine Department, Instituto de Investigación Sanitaria La Fe (IIS La Fe)/IVI-RMA Global Research Alliance, IVI Foundation, Valencia, Spain

**Keywords:** extracellular vesicles, endometriosis, eutopic endometrium, infertility, pathogenesis, pregnancy disorders

## Abstract

**STUDY QUESTION:**

Could the miRNA cargo of extracellular vesicles (EVs) secreted by eutopic endometrium from women with endometriosis be involved in the pregnancy complications related to endometriosis?

**SUMMARY ANSWER:**

EVs secreted by eutopic endometrium from women with endometriosis present altered miRNA that modulates biological processes related to pregnancy disorders.

**WHAT IS KNOWN ALREADY:**

Communication between endometrial cells, immune cells, endothelial cells, and the embryo is essential for the correct establishment and development of pregnancy, and EVs have been proposed as key mediators of this dialogue. This study aimed to elucidate whether EV-mediated communication is altered in endometriosis, by analysing the miRNA cargo of EVs secreted by eutopic endometrium from women with endometriosis, after differentiation to the gestational phase, compared with the miRNA cargo of EVs secreted by eutopic endometrium from healthy women, also after differentiation to the gestational phase.

**STUDY DESIGN, SIZE, DURATION:**

This was a prospective experimental comparative study in which endometrial organoids were derived from eutopic endometrium of patients with endometriosis (n = 16) and healthy women (n = 15).

**PARTICIPANTS/MATERIALS, SETTING, METHODS:**

Organoids were differentiated to mimic the gestational phase through supplementation with pregnancy hormones. EVs were then isolated from the culture medium, characterized, and their miRNA cargo was profiled by sequencing. Target genes of the significantly differentially expressed miRNAs were identified, and functional enrichment analysis was subsequently performed. Finally, the organoids were co-cultured with human endometrial stromal cells (hESCs) and human placental choriocarcinoma JAR cells, and the effects of EV-derived miRNAs were assessed by qPCR using representative target genes selected based on their roles in endometrium–embryo communication and epithelial–mesenchymal and mesenchymal–epithelial transitions (EMT–MET) in organoids, EMT in stromal (hESC) cells, and endometrium–embryo communication in JAR cells.

**MAIN RESULTS AND THE ROLE OF CHANCE:**

EVs were successfully isolated. miRNA-seq showed eight significantly differentially expressed miRNAs (false discovery rate [FDR] < 0.05); six were downregulated: miR-1290, miR-1246, miR-320d, miR-4516, miR-12136, and miR-3065-5p; and two were upregulated: miR-191-5p, and miR-335-5p. These miRNAs target 3964 genes and GO enrichment analysis identified 789 biological processes (FDR < 0.05), mainly involved in embryo development and developmental growth, immune system function, angiogenesis, and epithelial–mesenchymal transition. KEGG pathway analysis revealed 32 deregulated pathways (FDR < 0.05), including the PI3K–Akt pathway commonly related to the pathogenesis of preeclampsia. Validation in gestational epithelial endometriosis organoids showed upregulation of *IGF2* and *NR2F2*, and downregulation of *LIF*. Within the EMT–MET group*, PTK2B* was downregulated, whereas *WASF3* and *RAC1* were upregulated. In hESC cells co-cultured with gestational epithelial endometriosis organoids, *SFRP1*, *CDC42*, *RAC1*, and *WASF3* were upregulated, while *PTK2B* was downregulated, reflecting the miRNA cargo of ENDO-GEST-derived EVs. Similarly, in JAR cells co-cultured with gestational epithelial endometriosis, *NR2F2* and *IGF2* were upregulated, whereas *MDFI*, *SP3*, and *RDH10* were downregulated. All results were consistent with the inverse expression patterns of their regulatory miRNAs in secreted EVs.

**LARGE SCALE DATA:**

All raw sequencing data are available through the Gene Expression Omnibus (GEO) under accession number GSE302961.

**LIMITATIONS, REASONS FOR CAUTION:**

This was an *in vitro* study in which the conditions of the endometrial organoid culture could not fully replicate the intrauterine environment. In addition, due to the limited amount of RNA obtained from EVs, sample pooling was required for downstream analyses, which may have reduced our ability to assess inter-sample variability.

**WIDER IMPLICATIONS OF THE FINDINGS:**

EVs secreted by eutopic endometrium from women with endometriosis present altered miRNA that modulates processes essential for a successful pregnancy and could serve as potential diagnostic biomarkers for endometriosis and pregnancy disorders. These results also open avenues for further research about potential targets to promote pregnancy success in women with endometriosis.

**STUDY FUNDING/COMPETING INTEREST(S):**

This study was supported by the Spanish Ministry of Education through FPU (FPU21/00988 awarded to A.M.-P.), the Carlos III Health Institute, and cofounded by the European Social Fund (ESF) ‘Investing in your future’ through PI21/00184 and PI24/00961 (awarded to H.F.) and F122/00102 (awarded to A.B.-R.), a Sara Borrell Contract (CD23/00157 awarded to A.C. and CD24/00184 awarded to A.V.), and Miguel Servet Programme grants (CP20/00120 awarded to H.F. and CP19/00149 awarded to I.C.). This study was also supported by Fundació La Marató de TV3 (12/C/2024). The authors have no conflicts of interest to disclose.

WHAT DOES THIS MEAN FOR PATIENTS? Endometriosis is associated with a higher risk of pregnancy loss and complications. A successful pregnancy depends on proper communication between the embryo and the lining of the uterus. Small particles called extracellular vesicles help with this communication by carrying molecular ‘messages’ that influence other cells. In this study, laboratory models of the uterine lining during early pregnancy showed that these messages are altered in women with endometriosis, potentially contributing to problems with implantation and early pregnancy development. These messages include microRNAs, which are small molecules that help control how genes are expressed. Changes in these molecules could be used as biomarkers to support earlier and more accurate diagnosis of endometriosis, especially in women with fertility problems. They may also serve as targets for new treatments aimed at improving fertility and pregnancy outcomes.

## Introduction

Endometriosis is one of the most common gynaecological disorders worldwide, affecting ∼5–10% of women of reproductive age. This chronic disease is characterized by the abnormal presence of endometrial tissue outside the uterus, accompanied by chronic inflammation (Lamceva *et al.*, 2023). Women with endometriosis often experience severe abdomen and pelvic pain, dyspareunia, dysmenorrhea and, in 30–50% of cases, infertility. Historically, pregnancy was seen as having a positive impact on endometriosis and its symptoms (Zullo *et al.*, 2017). However, more recent research suggests that endometriosis may influence pregnancy outcomes through various mechanisms, including disruptions in endocrine and inflammatory balance, molecular and functional abnormalities of the eutopic endometrium, impaired deep placentation, and the altered decidualization of endometriotic tissue (Zullo *et al.*, 2017; de Ziegler *et al.*, 2019; Pirtea *et al.*, 2021; Ibiebele *et al.*, 2022). This could explain the fact that endometriosis has recently been associated with increased risk of impaired embryo implantation, miscarriage, preeclampsia (PE), placenta previa, preterm birth, and small for gestational age (SGA) babies (Kobayashi *et al.*, 2020; Vercellini *et al.*, 2023; Gebremedhin *et al.*, 2024). However, the molecular mechanisms underlying these complications remain largely unknown, mainly due to the inaccessibility of uterine tissue during pregnancy, underscoring the need for reliable biomarkers and novel therapeutic approaches to improve reproductive outcomes in endometriosis.

Successful pregnancy requires a proper cross-talk between endometrial epithelial and stromal cells, immune cells, endothelial cells, and the embryo to ensure successful implantation and the formation of a functional maternal–foetal interface through the placenta (Kurian and Modi, 2019; Liu *et al.*, 2022). Maternal endometrial tissue plays a key role in intercellular signalling during early pregnancy, by sending paracrine signals to the embryo and nearby tissues, and autocrine signals to itself (Beal *et al.*, 2023). Recent studies have proposed extracellular vesicles (EVs) as mediators of this communication system (Chu *et al.*, 2024; Merino-Pérez *et al.*, 2025), suggesting an important role in supporting decidualization, endometrial receptivity, trophoblast adhesion and development, and blood vessel formation (Greening *et al.*, 2017; Beal *et al.*, 2023; Segura-Benítez *et al.*, 2023). EVs are cell-derived nano-sized (30–1000 nm) membranous structures, produced by all cell types, that transport proteins, mRNA, and other noncoding RNAs, even between distant cells (Van Niel *et al.*, 2018). Their EV cargos vary depending on the cell type of origin and the cellular physiological state (Kurian and Modi, 2019; Scheck *et al.*, 2022; Juárez-Barber *et al.*, 2023). Previously, we have described the altered microRNA (miRNA) cargo of endometrial EVs from women with adenomyosis (Juárez-Barber *et al.*, 2023). Other studies have also described EVs containing miRNAs in the endometrium from women with endometriosis (Scheck *et al.*, 2022; Chu *et al.*, 2024); such EVs could potentially disrupt implantation-related processes and early pregnancy events. These findings highlight the clinical potential role of miRNAs from EVs as biomarkers and therapeutic targets to manage pregnancy complications in endometriosis.

miRNAs are short (22 nucleotides-long) non-coding RNA molecules that can act as gene regulators by inhibiting translation or inducing mRNA degradation (Liu *et al.*, 2020). miRNAs regulate hundreds of protein-coding genes, acting as key transcriptional regulators for many biological processes, including fertility-related functions as pregnancy process (Kozomara *et al.*, 2019; Tan *et al.*, 2019). Based on this, we hypothesized that the miRNA cargo in EVs secreted by the eutopic endometrium of women with endometriosis may be altered during gestation, contributing to the adverse pregnancy outcomes observed in these patients. Therefore, this study aimed to analyse the miRNA content of EVs secreted by organoids derived from eutopic endometrium of women with endometriosis differentiated to the early gestational phase, to explore their role in pregnancy complications and identity potential diagnostic biomarkers and therapeutic targets that could improve early detection, prevention, and management of these conditions. The organoid model recapitulates the functional and morphological features of the original tissue, enabling the study of the role of EVs in pregnancy, while overcoming ethical and technical constraints of obtaining endometrial biopsies from pregnant women.

## Materials and methods

### Patient samples

Since the eutopic endometrium, and more specifically, the epithelial cells of this eutopic endometrium, are in direct contact with the embryo and exerts an immediate and functional influence on implantation and pregnancy outcomes, we collected biopsies from eutopic endometrium by hysteroscopy (Karl Storz SE & Co. KG, Tuttlingen, Germany) from Caucasian women (aged 18 ≤ 40 years; BMI ≤ 30 kg/m^2^) with endometriosis (16 patients/group) and without endometriosis (15 patients/group) at Hospital La Fe and the IVI Valencia Clinic (Supplementary Table S1). Patients with any other suspected or diagnosed uterine pathologies, such as uterine fibroids or adenomyosis, were excluded. Women with endometriosis were diagnosed with either stage 3 or 4 disease, and they had all experienced at least 6 months of infertility. Healthy oocyte donors with a standard uterine volume and no evidence of endometriotic lesions were selected as controls. Patients were free from other gynaecological pathologies and had not used any medications in the 3 months prior. The collection and use of human tissue were approved by the Clinical Research Ethics Committee at Hospital La Fe (2106-FE-069-HF, 27 February 2023). Informed written consent was obtained from all the participants.

### Establishment of endometrial organoids and their differentiation into gestational phase

The endometriosis and control endometrial organoids were derived from eutopic endometrium and differentiated into gestational phase as previously described (Boretto *et al.*, 2017; Turco *et al.*, 2017). Briefly, endometrial biopsies from patients with endometriosis (n = 16) and healthy patients (n = 15) were mechanically and enzymatically digested with 50 U/ml Dispase II (D4693) and 4 mg/ml collagenase-V (C9263; both from Sigma-Aldrich, St. Louis, MO, USA) to isolate the epithelial glandular fractions. Glandular elements were retained by filtering the supernatant through 100-µm cell sieves (431752; Corning, Tewksbury, MA, USA). The pellet was resuspended in 15% DMEM/F12 (12634010; Invitrogen, Paisley, UK) and 85% Matrigel (354234; Corning, Bedford, MA, USA). Organoids were seeded in 20 µl droplets and cultured with 250 µl of expansion medium ExM (Turco *et al.*, 2017); ExM was replaced every 2 days; and the organoids were passaged every 4–7 days. For gestational-phase differentiation, after 4 days in ExM, endometriosis (ENDO-GESTorg) and control organoids (CONTROL-GESTorg) were treated with 10 nM oestrogen (E2), 1 µM progesterone (P4), and 1 µM cyclic adenosine monophosphate (cAMP), with an additional 20 ng/ml of prolactin (PRL; PeproTech, Cranbury, NJ, USA, 100-07) and 20 ng/ml human placental lactogen for 8 days (hPL; R&D, Minneapolis, MN, USA, 5757-PL). The derivation and differentiation of the ENDO-GESTorg and CONTROL-GESTorg were also corroborated as previously described (Boretto *et al.*, 2017; Turco *et al.*, 2017; Juárez-Barber *et al.*, 2023). Expression of MUC-1, Ki67, pan-cytokeratin, and laminin, as well as Periodic acid–Schiff (PAS) staining confirmed the epithelial origin of the cells (Supplementary Fig. S1), while α-tubulin and SOX-9 expression corroborated the differentiation of the organoids into gestational phase (Supplementary Fig. S2).

### EV isolation by ultracentrifugation

After organoid differentiation, conditioned medium was collected from the ENDO-GESTorg cultures (n = 16) and pooled in four different aliquots (n = 4 patients/pool), while conditioned medium was collected from CONTROL-GESTorg cultures (n = 15) and pooled in three different aliquots (n = 5 patients/pool), due to the limited RNA amount from individual EV samples. EVs were then isolated by ultracentrifugation. Briefly, pools were first centrifuged at 300 *g* for 10 min at 4°C, followed by 2000 *g* for 20 min at 4°C to pellet the cells. The supernatant was then transferred to polycarbonate tubes and centrifuged at 10 000 *g* for 30 min at 4°C to remove cellular debris. Finally, EVs (ENDO-GESTevs/CONTROL-GESTevs) were isolated by double ultracentrifugation at 100 000 *g* for 70 min at 4°C using an Avanti J-30i centrifuge (Beckman-Coulter, Brea, CA, USA) with a JA30.50 Ti rotor. After the supernatant was discarded, EVs were resuspended in 30 µl of PBS; 10 µl was used for characterization studies by nanoparticle tracking analysis (NTA), Exoview, and transmission electron microscopy (TEM); the remaining EVs were used for miRNA extraction and subsequent miRNA-seq analysis.

### Nanoparticle tracking analysis

The size distribution and concentration of EVs were assessed using NTA on a NanoSight NS300 instrument (Malvern Panalytical, Madrid, Spain). The samples were diluted in PBS at a 1:8 ratio and two replicates were measured.

### Transmission electron microscopy

The size distribution and morphology of the EVs were analysed using a FEI Tecnai G2 Spirit BioTwin TEM (Thermo Fisher Scientific, Waltham, MA, USA). For this purpose, 6 µl of EVs suspended in PBS were placed on a carbon-coated grid and stained with 2% uranyl acetate.

### Immunofluorescent tetraspanin staining with the Exoview kit

The presence of CD9, CD63, and CD81 tetraspanins on the surface of isolated EVs was analysed using the Exoview R100 Kit (Nanoview Bioscience, Boston, MA, USA; Wise *et al.*, 2021; Deng *et al.*, 2022). The Exoview Tetraspanin Chip captures the presence of tetraspanins using three distinct spots, each employing one of the tetraspanins as a capture probe. Microarray chips coated with antibodies targeting CD9, CD63, and CD81 were pre-scanned following the manufacturer’s instructions. Each sample was diluted 1:100 in Incubation Solution 1X and 50 µl of the diluted sample per assay was incubated overnight at room temperature on an Exoview Tetraspanin Chip and were subsequently washed four times. Next, 250 µl of the fluorescent antibody mixture was added to each chip and incubated for 1 h at room temperature in the dark. Finally, the chips were washed in the provided buffers, dried, and imaged using an ExoView R100 platform. Data were then exported using Exoview Analyzer version 3.2 (NanoView Biosciences).

### miRNA extraction and library construction

miRNA was isolated from ENDO-GESTevs and CONTROL-GESTevs using an miRNeasy Serum/Plasma Kit (Qiagen, Germantown, MD, USA) according to the manufacturer’s instructions. miRNA NGS libraries were generated using a QIAseq miRNA Library Kit (Qiagen). Briefly, after adapter ligation, unique molecular identifiers (UMIs) were introduced in the reverse transcription step, and cDNA was amplified using PCR (22 cycles) with subsequent sample purification. Library preparation was quality-controlled using capillary electrophoresis (Tape D1000). Based on the quality of the inserts and the concentration measurements, the libraries were pooled in equimolar ratios (n = 4 samples/pool of ENDO-GESTorg and n = 3 samples/pool of CONTROL-GESTorg). The library was sequenced on a NextSeq 2000 sequencing instrument (Illumina Inc, San Diego, CA, USA) using a single-end 75-base pair read and an additional 6-base pair index read according to the manufacturer’s instructions.

### miRNA-sequencing data process

The library data were processed and statistically analysed with R/Bioconductor (v3.5.0). Raw data were de-multiplexed and FASTQ files for each sample were generated using bcl2fastq2 software v2.20.0.422 (Illumina Inc.). Sequencing samples yielded an average of 10 million reads per sample. The Empirical Analysis of DGE algorithm from the CLC Genomics Workbench (v23.0.5) was used for differential expression analysis applying the default settings. This is an implementation of the exact test for two-group comparisons incorporated in the EdgeR Bioconductor package (Love *et al.*, 2014). Each demultiplexed sample is delivered with a pair of FASTQ files whose expected read structure is depicted in Supplementary Fig. S3.

The 19-nucleotide common sequence was: AACTGTAGGCACCATCAAT. Differentially expressed miRNAs (DEmiRNAs) were considered significant when the *P*-value adjusted by the false discovery rate (FDR) < 0.05. For all unsupervised analyses, only miRNAs with at least 10 counts summed over all samples were considered. A variance-stabilizing transformation was performed on the raw count matrix using the vst function from the DESeq2 package (v1.28.1) in R. All raw sequencing data are available through the Gene Expression Omnibus (GEO) under accession number GSE302961. The miRNAs with the highest variance were used for the principal component analysis (PCA). The variance was calculated agnostically to the pre-defined groups (blind = TRUE). A variance-stabilized transformation was performed on the raw count matrix and the 35 miRNAs with the highest variance across samples were selected for hierarchical clustering.

### miRNA target prediction and functional enrichment analyses

The target genes of the DEmiRNAs were first predicted by the microRNA Data Integration Portal (mirDIP). A Venn diagram was constructed to compare the target lists of each miRNA individually with the target list generated when all the miRNAs were analysed together as a single group. The targets found in the combined group were merged with those that were exclusively present in any of the individual groups. The same process was applied using the experimentally validated miRNA-target interactions database, miRTarBase. The results from both databases were integrated, and duplicates were removed. Next, Gene Ontology (GO) enrichment analysis was carried out with the resulting targets via G Profiler. The most significant biological processes with an FDR < 0.05 were considered enriched functions regulated by the miRNA cargo of the EVs. A chord plot showing the relationship between GO functional groups and miRNAs was plotted using an online bioinformatics tool (www.bioinformatics.com.cn).

### Validation of miRNA expression

To validate the expression of DEmiRNAs contained in ENDO-GESTevs, we selected the DEmiRNAs that regulate the most relevant target genes within the crucial enriched functional groups. miRNAs were isolated individually from each pool using the miRNeasy Serum/Plasma Kit (Qiagen) according to manufacturer’s instructions, from CONTROL-GESTevs and ENDO-GESTevs. miRNA expression was quantified in each pool individually using miRCURY LNA miRNA PCR Assays (Qiagen), following the manufacturer’s instructions. Reverse transcription was performed with a miRCURY LNA RT Kit using 10 ng of total RNA per reaction. Quantitative PCR of these seven DEmiRNAs was carried out on a LightCycler 480 system (Roche, Basel, Switzerland) using a miRCURY LNA SYBR Green PCR Kit (Qiagen). Relative expression levels were calculated using the ΔCt method. UniSp100 and UniSp101 were used as spike-in controls, while miR-16-5p and let-7i-5p were selected as endogenous controls because they showed the most stable expression levels across the samples. Normalization was performed using the mean expression value of miR-16-5p and let-7i-5p.

### HESC and JAR culture

Human endometrial stromal cells (hESC, ATCC CRL-4003) and JAR choriocarcinoma cells (ATCC HTB-144) were thawed and seeded into T-75 flasks. hESCs were maintained in stromal medium consisting of DMEM/F12 with GlutaMAX™ (Thermo Fisher Scientific) supplemented with 10% heat-inactivated foetal bovine serum (Gibco, Grand Island, NY, USA; 17934731), 1% penicillin/streptomycin (Fisher Scientific, 11548876), and 1% amphotericin B (Merck, A2942-50ML). JAR cells were cultured in RPMI 1640 medium (Thermo Fisher Scientific, 11530586) supplemented with 10% foetal bovine serum, 2.5 g/l glucose (Merck, 1083469029), 1 mM sodium pyruvate (Cymit Química, Barcelona, Spain; 3B-P0582-25g), 10 mM HEPES (Gibco, 12509079), gentamicin (Gibco, 15820243), and Fungizone (Thermo Fisher Scientific, 15290067). Cultures were maintained at 37°C in a humidified atmosphere containing 5% CO_2_ and monitored until reaching ∼80–90% confluence. For passaging, cells were detached using trypsin (Gibco, 11588846), centrifuged at 600 *g* for 5 min, and reseeded into fresh T-75 flasks containing the appropriate complete medium.

### Establishment of the co-culture using Transwell inserts

To establish the co-culture system, 12-well plates designed for Transwell insert compatibility (Falcon, Corning, NY, USA 353503) were used. The selected Transwell inserts contained a 0.4 µm porous membrane (Falcon, 353180). On Day 0, endometrial organoids derived from six ENDO-GESTorg and six CONTROL-GESTorg were used for each condition. For each woman, two Matrigel droplets (25 µl each) were seeded onto the Transwell insert for co-culture with hESCs, and two additional droplets per women were seeded for co-culture with JAR cells, resulting in four droplets per women. Organoids were embedded in Matrigel with 250 µl of ExM. On the same day, either hESCs or JAR cells were plated as a monolayer at the bottom of the 12-well plate (100 000 cells per well). Once the cells had adhered to the well surface, the Transwell insert containing the organoid droplets was placed on top, and 1 ml of ExM medium was added per well. After 4 days, the medium was replaced with ExM supplemented with 10 nM oestradiol (E2), 1 µM progesterone (P4), and 1 µM cyclic adenosine monophosphate (cAMP), with the addition of 20 ng/ml prolactin (PRL; PeproTech, Cranbury, NJ, USA, 100-07) and 20 ng/ml human placental lactogen (hPL; R&D Systems, Minneapolis, MN, USA, 5757-PL) to induce gestational-phase differentiation. The porous membrane allows the passage of EVs and soluble factors between the organoids and co-cultured cells throughout the experiment. The co-culture was maintained for 8 days following medium replacement. At the end of the experiment, all cells were collected to assess the effects of EV-derived microRNAs on their gene expression. The co-culture scheme has been added as Supplementary Fig. S4.

### Validation of gene expression

To assess autocrine regulation by the endometriotic organoids, the expression of genes targeted by the miRNAs contained in ENDO-GEST EVs was evaluated by individually extracting RNA from ENDO-GESTorg and CONTROL-GESTorg of each patient included in the study (n = 7 per group). To evaluate paracrine regulation exerted by endometrial epithelial cells of the organoids on stromal cells and the embryo, RNA was extracted from hESCs and JAR cells that had been previously co-cultured with ENDO-GESTorg and CONTROL-GESTorg (n = 6 per group). Total RNA was extracted using an miRNeasy Mini Kit (Qiagen), according to the manufacturer’s instructions. Complementary DNA (cDNA) was synthesized from 500 ng of total RNA using the PrimerScript RT reagent kit (Takara, Kusatsu, Japan). Gene expression was analysed by quantitative real-time PCR (q-RT-PCR) using Power SYBR Green Master Mix (ThermoFisher) on a StepOnePlus system (Applied Biosystems, Waltham, MA, USA). Reactions were performed in technical triplicates, with an initial denaturation at 95°C for 10 min, followed by 40 cycles of 95°C for 15 s and 60°C for 1 min. Melting curve analysis was performed at the end of each run to confirm amplification specificity Relative gene expression levels were calculated using the 2^−ΔΔCt^ method, normalizing target gene expression to *GAPDH* as a housekeeping gene. For the organoids, which consist of endometrial epithelial cells, we selected genes from both the endometrium–embryo communication group, as they constitute the first layer of contact with the embryo, and the EMT–MET group, because these cells undergo phenotypic changes when displaced by the invading trophoblast. based on literature evidence (*IGF2, NR2F2, PTK2B, WASF3, LIF*, and *RAC1*). For the hESC cells, we evaluated the expression of some of the most highly represented genes from the EMT–MET group *(SFRP1, CDC42, PTK2B, RAC1*, and *WASF*3), as during the embryonic invasion process, endometrial stromal cells switch from a fibroblast-like phenotype to a more epithelioid or polygonal phenotype and organize into a dense cellular matrix (the decidua) that surrounds the implantation site, acting as a cushion and physical regulatory barrier against the invading trophoblast. In JAR cells, a trophoblastic cell line model, we assessed genes from the endometrium–embryo communication group (*NR2F2*, *SP3*, *MDFI*, *IGF2*, and *RDH10*). The sequences of all primers used for the analysed genes are provided in Supplementary Table S2.

### Statistical analysis

Omics data were analysed using R (version 3.5.1) and visualized using R core package (R Core Team, 2018), the gplots and ggplot2 packages, and GraphPad Prism 8.0 (GraphPad Software, San Diego, CA, USA). GraphPad Prism 8.0 was used for miRNA and gene expression validation analyses, applying Student’s *t*-tests, Wilcoxon tests, or Mann–Whitney based on whether the variables were parametric or non-parametric, according to the distribution of the data; *P *< 0.05 was considered statistically significant.

## Results

### Characterization of EVs secreted by gestational endometriosis organoids

**Figure 1.**
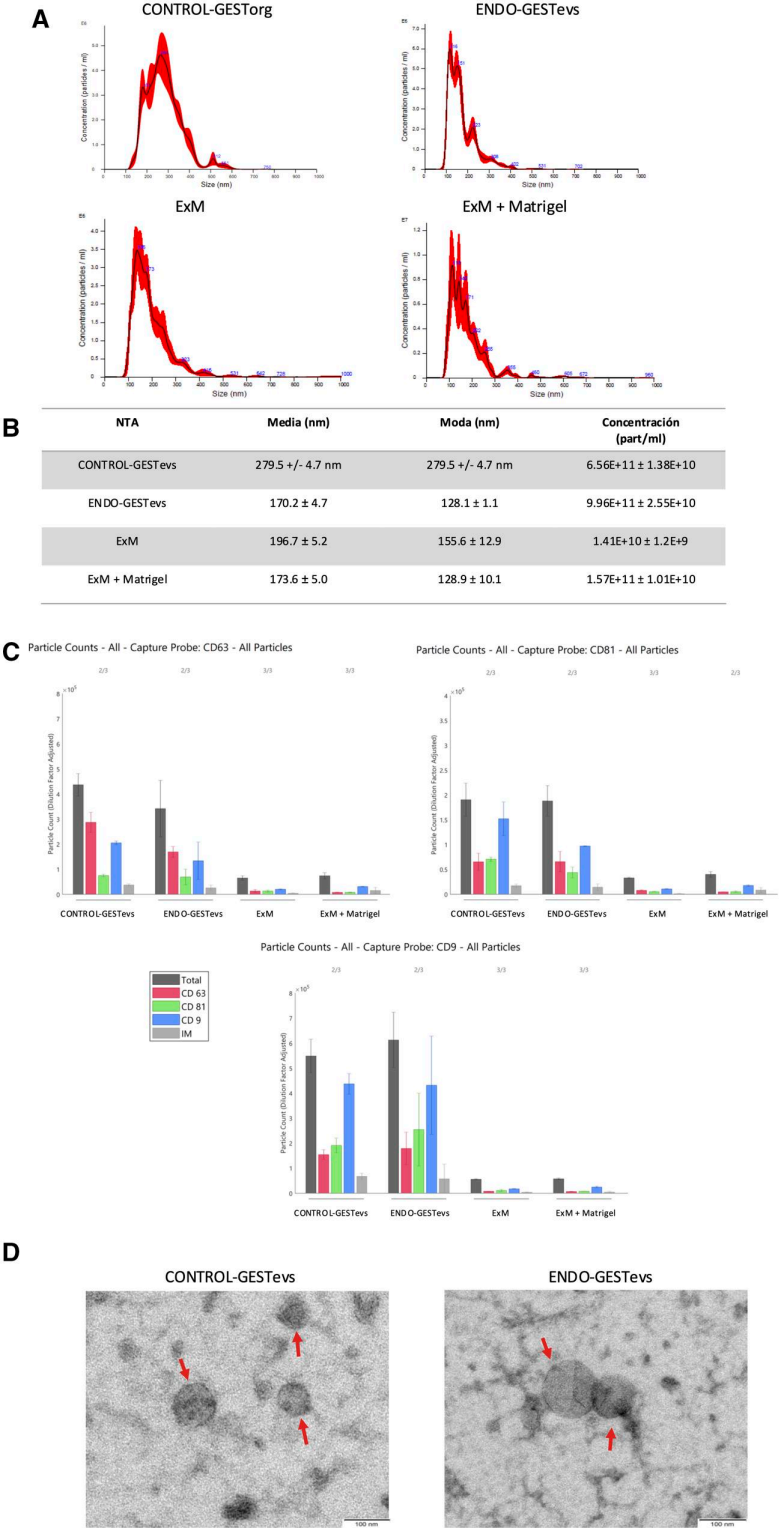
**Characterization of extracellular vesicles secreted by endometrial organoids generated from biopsies from women with gestational endometriosis versus healthy controls.** (**A**) Nanoparticle tracking analysis (NTA) of the size and concentration of nanoparticles isolated from conditioned medium from gestational endometrial organoids made from biopsies from healthy women (CONTROL-GESTorg), patients with endometriosis (ENDO-GESTorg), Fresh Median (ExM), and Matrigel-only conditioned media (ExM + Matrigel). (**B**) Summary table of the NTA results presenting the mean, mode size, and concentration values with their standard deviations (*SD*s). (**C**) Expression levels of the protein extracellular vesicle (EV) markers. CD63, CD81, and CD9 expression at the three different spots captured. (**D**) Representative image of the EV morphology of CONTROL-GESTevs and ENDO-GESTevs captured by transmission electron microscopy (TEM) as well as the size range of EVs visualized by TEM. The scale bar represents 100 nm.

Particles secreted by the ENDO-GESTorg and CONTROL-GESTorg organoids into the culture media were characterized by NTA, ExoView, and TEM. NTA showed a nanoparticle size between 100 and 300 nm and a concentration of 9.96E+11 ± 2.55E+10 (*SD*) particles/ml and 6.56E+11 ± 1.38E+10 (*SD*) in ENDO-GESTorg and CONTROL-GESTorg, respectively (Fig. 1A and B). NTA was also performed in non-conditioned culture media and Matrigel-only conditioned media to verify that the isolated EVs did not originate from the culture medium or Matrigel. In these control samples, NTA showed nanoparticles of between 100 and 200 nm and a concentration of 1.41E+10 ± 1.2E+9 particles/ml and 1.57E+11 ± 1.01E+10 particles/ml, respectively (Fig. 1A and B).

To verify whether the nanoparticles detected by NTA were EVs, a subsequent Exoview assay was performed. Transmembrane protein markers CD63, CD81, and CD9 were detected in nanoparticles obtained from ENDO-GESTorg and CONTROL-GESTorg conditioned culture media, while EVs were not found in non-conditioned culture media or Matrigel-only conditioned media (Fig. 1C). Finally, TEM imaging of the ultrastructure of the particles verified their distinctive cup-shaped morphology and a size range consistent with EVs, further confirming their presence (Fig. 1D).

### Global transcriptomic behaviour of the miRNA cargo from gestational endometriosis organoid EVs

**Figure 2.**
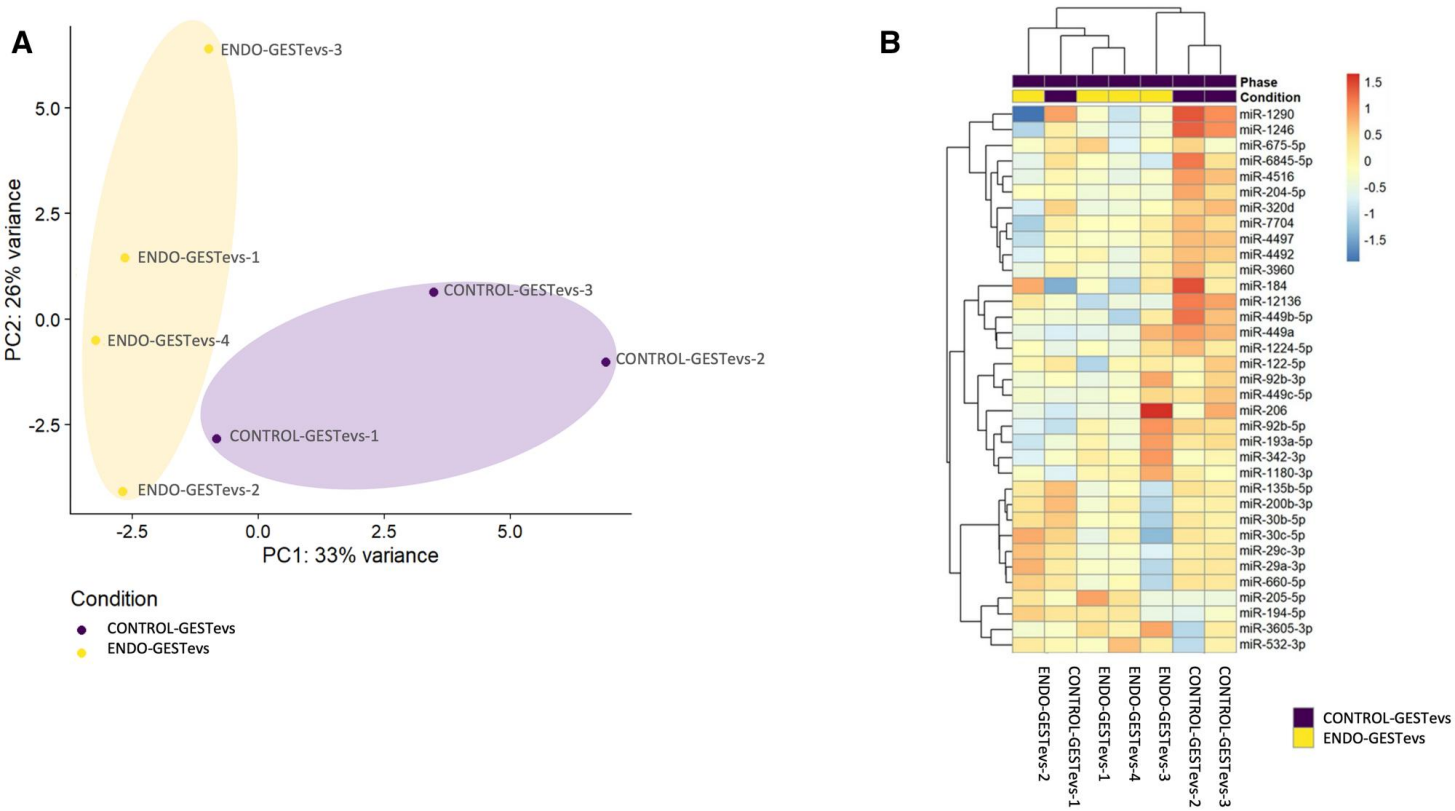
**The global transcriptomic behaviour of the micro (mi) RNA cargo of gestational endometriosis organoid extracellular vesicles compared with those derived from healthy controls.** (**A**) A principal component analysis (PCA) plot of the global transcriptome after normalization. The miRNAs with the highest variance were used for analysis. The variance was calculated agnostically to the pre-defined groups (blind = TRUE). (**B**) A heatmap representing the fold-enrichment score of miRNAs after unsupervised clustering. Each row represents one miRNA and each column represents one sample. The colour represents the difference in the count value to the row mean. ENDO-GESTevs pools are represented in yellow, while CONTROL-GESTevs pools are represented in purple. ENDO-GESTevs, Extracellular vesicles secreted by gestational endometriosis organoids; CONTROL-GESTevs, Extracellular vesicles secreted by gestational control organoids.

The miRNA cargo of ENDO-GESTevs/CONTROL-GESTevs was characterized by miRNA sequencing. PCA revealed distinct miRNA expression behaviour between the endometriosis and control samples (Fig. 2A). The hierarchically clustered heatmap of 35 miRNAs with the highest variance also showed different expression patterns between ENDO-GESTevs and CONTROL-GESTevs (Fig. 2B), corroborating the differential miRNA expression behaviour observed in the PCA.

### Differential miRNA expression of gestational endometriosis organoid EVs

**Figure 3.**
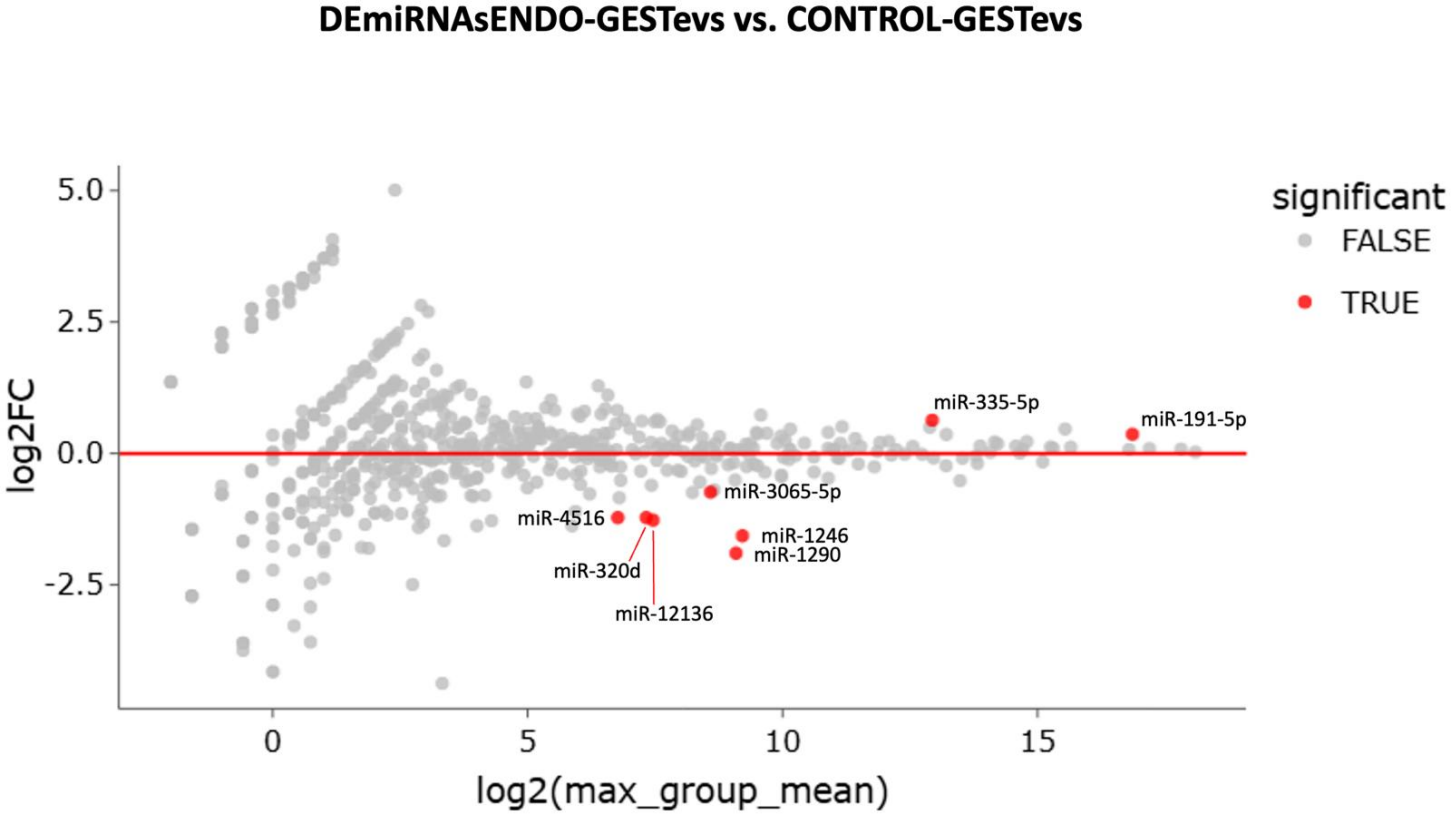
**Analysis of differentially expressed micro (mi) RNAs in gestational endometriosis organoid extracellular vesicles.** A minus-average plot representing differentially expressed miRNAs. All miRNAs significantly differentially expressed between the experimental and control groups are marked in red. Significant changes are defined as a false discovery rate (FDR) < 0.05. Upregulated miRNAs are shown above the *X*-axis, while downregulated miRNAs are shown below the *X*-axis. The *Y*-axis represents the log2 fold change (log2 FC). DEmiRNAs, differentially expressed miRNAs; ENDO-GESTevs, Extracellular vesicles secreted by gestational endometriosis organoids.

Deep sequencing analysis identified 289 DEmiRNAs between ENDO-GESTevs and CONTROL-GESTevs, with 8 of these being deemed significant (FDR < 0.05; Supplementary Table S3). Among these miRNAs, six were downregulated: miR-1290 (log2 fold change [FC] = −1.89), miR-1246 (FC = −1.56), miR-320d (FC = −1.21), miR-4516 (FC = −1.22), miR-12136 (FC = −1.26), and miR-3065-5p (FC = −0.73); in turn, two miRNAs were upregulated: miR-191-5p (FC = 0.36) and miR-335-5p (FC = 0.63; Fig. 3). Specifically, seven of them (miR-1290, miR-1246, miR-4516, miR-12136, miR-3065-5p, miR-191-5p, and miR-335-5p) had been previously described as being related to adverse pregnancy outcomes.

### Global gene regulation by miRNA cargo in gestational endometriosis organoid EVs

To predict the target genes of the DEmiRNAs, we first used mirDIP, a comprehensive database that consolidates human microRNA−gene interactions from multiple sources. Additionally, we utilized miRTarBase, a database that compiles experimentally validated miRNA-target interactions. In the mirDIP database, only target genes classified as very high confidence, corresponding to the top 1% based on the score class, were considered. This led to the identification of 1216 target genes, while miRTarBase provided 2927 target genes. The integration of both databases resulted in 3964 final target genes (Supplementary File S1).

### Functional implications of the miRNA cargo target genes in the gestational endometriosis organoid EVs

Functional enrichment analysis of 3964 target genes identified a total of 789 biological processes significantly enriched in samples from patients with endometriosis. These were classified into distinct functional groups: in particular several were related to endometrium–embryo communication, the immune system, angiogenesis, and epithelial–mesenchymal and mesenchymal–epithelial transitions (EMT–MET) (Supplementary File S2). Additionally, other relevant processes included those associated with the cell cycle and apoptosis, cell communication and stimulus response, metabolic and biosynthetic processes, and cell signalling (Supplementary File S2).

**Figure 4.**
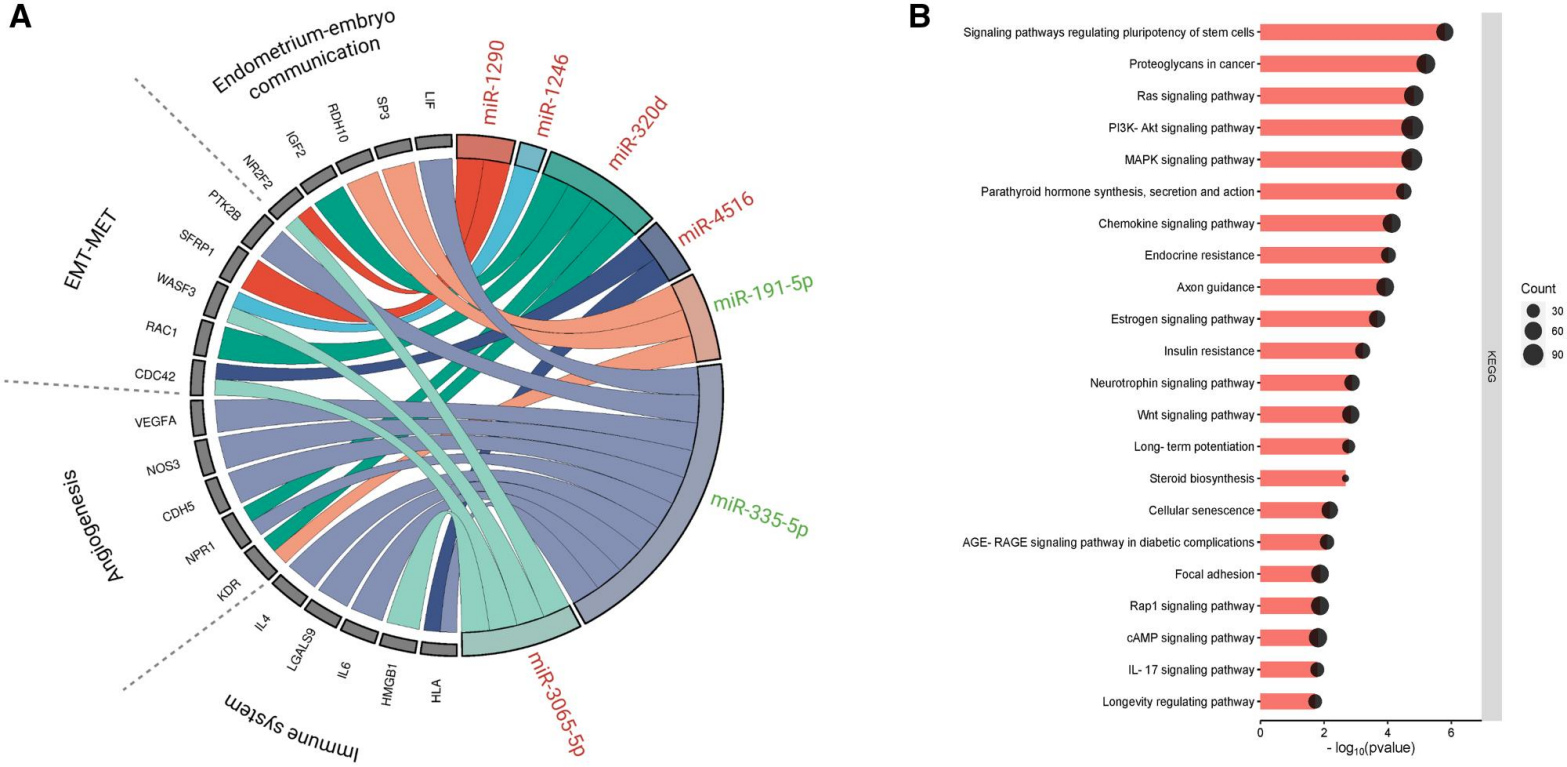
**Functional enrichment analysis of the predicted gene targets of the differentially expressed micro (mi) RNAs in gestational endometriosis organoid extracellular vesicles.** (**A**) Association of differentially expressed miRNAs in gestational endometriosis organoid extracellular vesicles with the most representative target genes involved in the highlighted functional groups. Upregulated miRNAs are represented in green, while downregulated miRNAs are represented in red. (Other colours in the chord plot are used solely for visual clarity and do not represent any biological meaning.). (**B**) Highlighted canonical KEGG pathways enriched by the target genes of miRNA cargo in ENDO-GESTevs. The *x*-axis represents statistical significance, and the circle size indicates the number of implicated genes. ENDO-GESTevs, Extracellular vesicles secreted by gestational endometriosis organoids.

Subsequently, the most representative target genes in each of the highlighted functional groups were studied (Fig. 4A). Among the target genes in the endometrium–embryo communication functional group, we would like to highlight: *LIF*, regulated by miR-335-5p; *SP3*, regulated by miR-191-5p; *RDH10*, targeted by miR-191-5p; *IGF2*, targeted by miR-320d; and *NR2F2*, targeted jointly by miR-3065-5p and miR-1290. Within the EMT–MET functional group, *PTK2B*, targeted by miR-335-5p; *SFRP1*, regulated by miR-1290; *RAC1*, targeted by miR-320d; *CDC42*, targeted by miR-3065-5p and miR-4516, and *WASF3*, controlled by miR-1246 and miR-3065-5p stood out. In addition, within the angiogenesis functional group, *VEGFA*, *NOS3*, and *CDH5*, targeted by miR-335-5p; and *NPR1* and *KDR*, targeted by both miR-335-5p and miR-320d. Finally, within the immune system group, we would like to draw attention to *IL4*, *LGALS9*, and *IL6*, targeted by miR-335-5p; *HMGB1*, targeted by miR-3065-5p; and *HLA*, targeted by both miR-4516 and miR-335-5p. The most relevant genes in the highlighted functional groups were regulated by miR-335-5p. However, none of them were regulated by miR-12136, so the latter can be considered the least relevant regulator. Meanwhile, KEGG pathway analysis revealed 32 deregulated pathways (FDR < 0.05), including the Wnt, Oestrogen, and PI3K–Akt signalling pathways, which are involved in implantation, decidualization, endometrial remodelling, and the pathogenesis of preeclampsia, and the Rap1 signalling pathway, which plays a role in decidualization (Fig. 4B).

### Validation of miRNA expression

**Figure 5.**
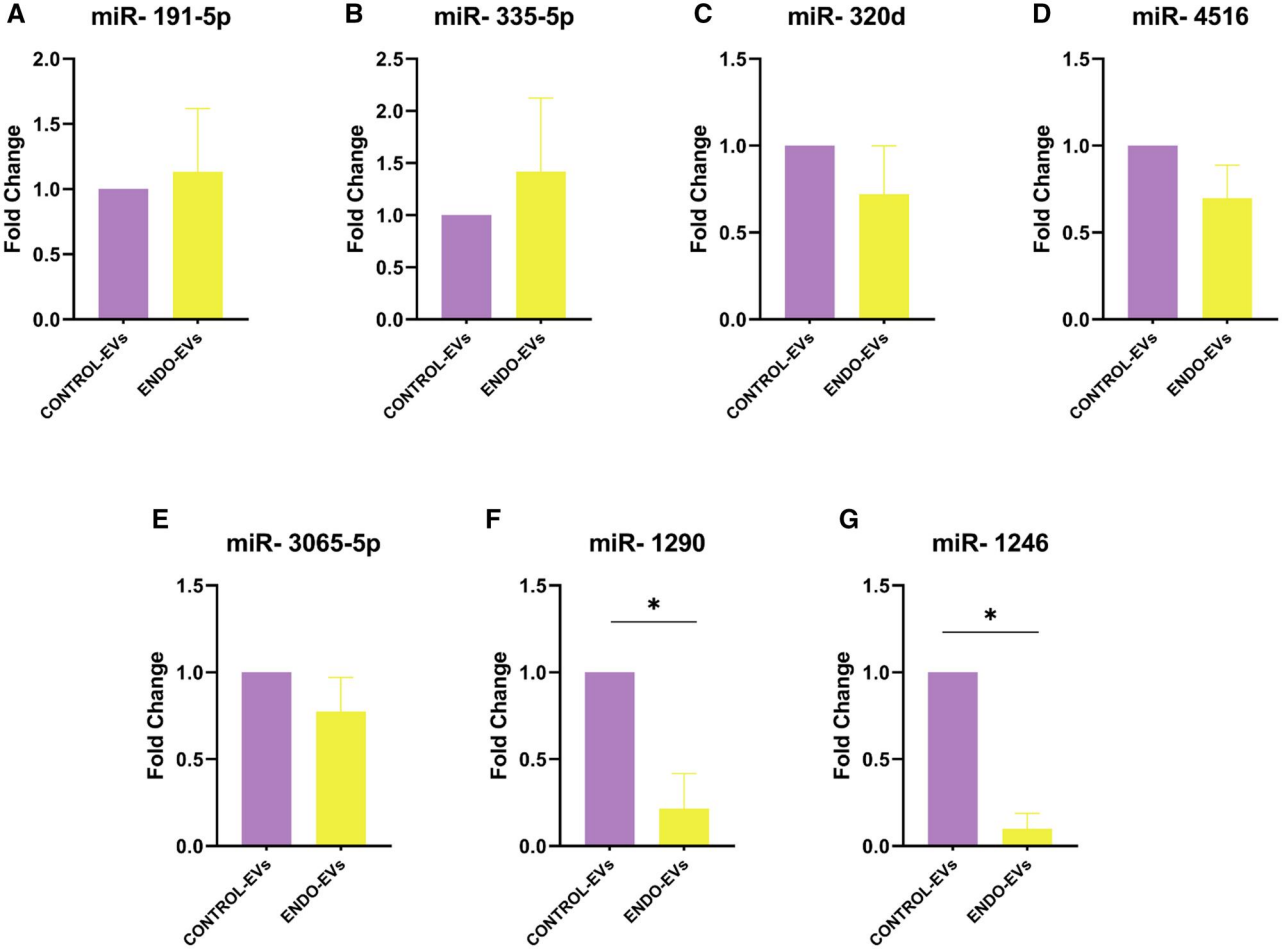
**Validation of micro (mi) RNA in extracellular vesicles.** Expression of (**A**) miR-191-5P, (**B**) miR-335-5p, (**C**) miR-320d, (**D**) miR-4516, (**E**) miR-3065-5p, (**F**) miR-1290, and (**G**) miR-1246 was validated in ENDO-GESTevs and compared with that in CONTROL-GESTevs groups by qRT-PCR. Relative expression levels were calculated using the ΔCt method. miR-16-5p and let-7i-5p were selected as endogenous controls because they showed the most stable expression levels across the samples. Normalization was performed using the mean expression value of miR-16-5p and let-7i-5p. Statistical analysis was performed using the Mann–Whitney *U*-test. *P < 0.05; **P < 0.005. ENDO-GESTevs, Extracellular vesicles secreted by gestational endometriosis organoids; CONTROL-GESTevs, Extracellular vesicles secreted by gestational control organoids.

To validate the results obtained from the miRNA-seq analysis, a subset of miRNAs was selected for experimental confirmation by quantitative PCR (qPCR). Specifically, we chose miRNAs predicted to regulate the most representative genes within the most relevant functional groups identified in the analysis. miR-191-5p and miR-335-5p were found to be upregulated in ENDO-GESTevs compared to CONTROL-GESTevs (FC=1.12 and 1.41, respectively) (Fig. 5A and B). In contrast, miR-320d, miR-4516, miR-3065-5p, miR-1290, and miR-1246 were downregulated in ENDO-GESTevs compared to CONTROL-GESTevs (FC= 0.71, 0.69, 0.77, 0.22, 0.09, respectively), with miR-1290 and miR-1246 showing statistically significant differences (*P *< 0.05) (Fig. 5C–G). The qPCR validation confirmed that all selected miRNAs exhibited expression patterns consistent with those observed in the sequencing data.

### Functional analysis of endometrial EV-miRNA in the autocrine regulation of epithelial organoids

**Figure 6.**
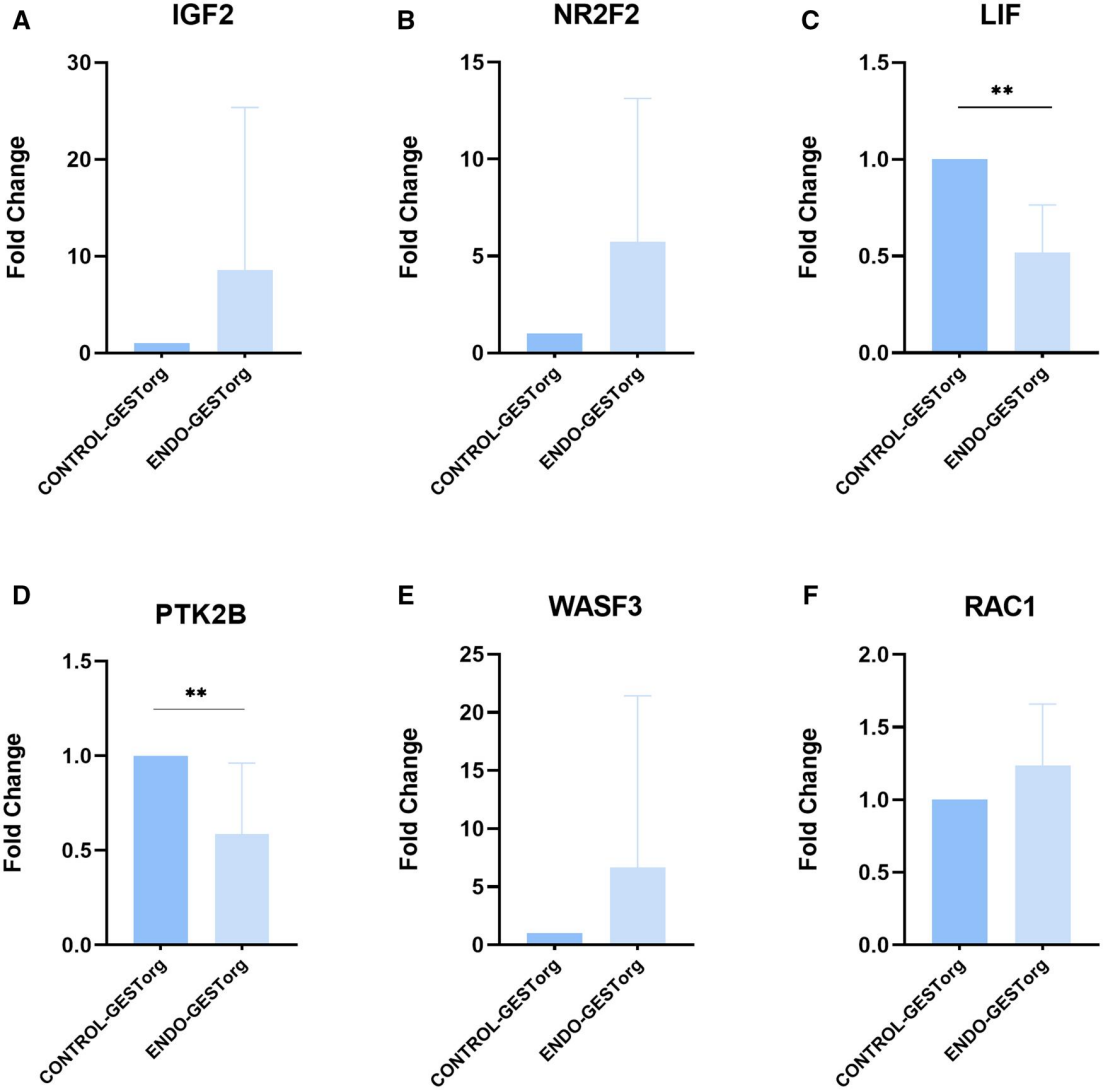
**Validation of micro (mi) RNA target genes in organoids.** Gene expression of (**A**) *IGF2*, (**B**) *NR2F2*, (**C**) *LIF*, (**D**) *PTK2B*, (**E**) *WASF3*, and (**F**) *RAC1* was validated in ENDO-GESTorg group and compared with that in the CONTROL-GESTorg group (n = 7 per group) by qRT-PCR. Relative gene expression levels were calculated using the 2^−ΔΔCt^ method, normalizing target gene expression to GAPDH as a housekeeping gene. Statistical analysis was performed using the Mann–Whitney *U*-test. *P < 0.05; **P < 0.005. ENDO-GESTorg, Gestational endometriosis organoids; CONTROL-GESTorg, Gestational control organoids.

From the most representative genes targeted by miRNAs contained in ENDO-GESTevs within the endometrial–embryo communication functional group, the validation performed in organoids (ENDO-GESTorg vs CONTROL-GESTorg) revealed that *IGF2* and *NR2F2* were significantly upregulated (FC = 8.56 and 5.75, respectively) (Fig. 6A and B). This upregulation is consistent with the observed downregulation of their corresponding regulatory miRNAs in EVs secreted into the ENDO-GESTorg culture medium: miR-320d for *IGF2*, and miR-3065-5p and miR-1290 for NR2F2. In contrast, *LIF* was downregulated (FC = 0.52; *P* < 0.01), in agreement with the upregulation of its regulatory miRNA, miR-335-5p, in the corresponding EVs (Fig. 6C). Within the EMT–MET functional group, *PTK2B* was downregulated in ENDO-GESTorg (FC = 0.58; *P* < 0.01) (Fig. 6D), correlating with the increased levels of its regulatory miRNA, miR-335-5p, in EVs. Conversely, *WASF3* was upregulated (FC = 6.67), consistent with the downregulation of its regulatory miRNAs, miR-1246 and miR-3065-5p, in the corresponding EVs (Fig. 6E). Similarly, *RAC1* was upregulated (FC = 1.24), in agreement with the decreased expression of its regulatory miRNA, miR-320d (Fig. 6F).

### Functional analysis of endometrial EV-miRNA in the paracrine regulation of stromal and trophectoderm cells

**Figure 7.**
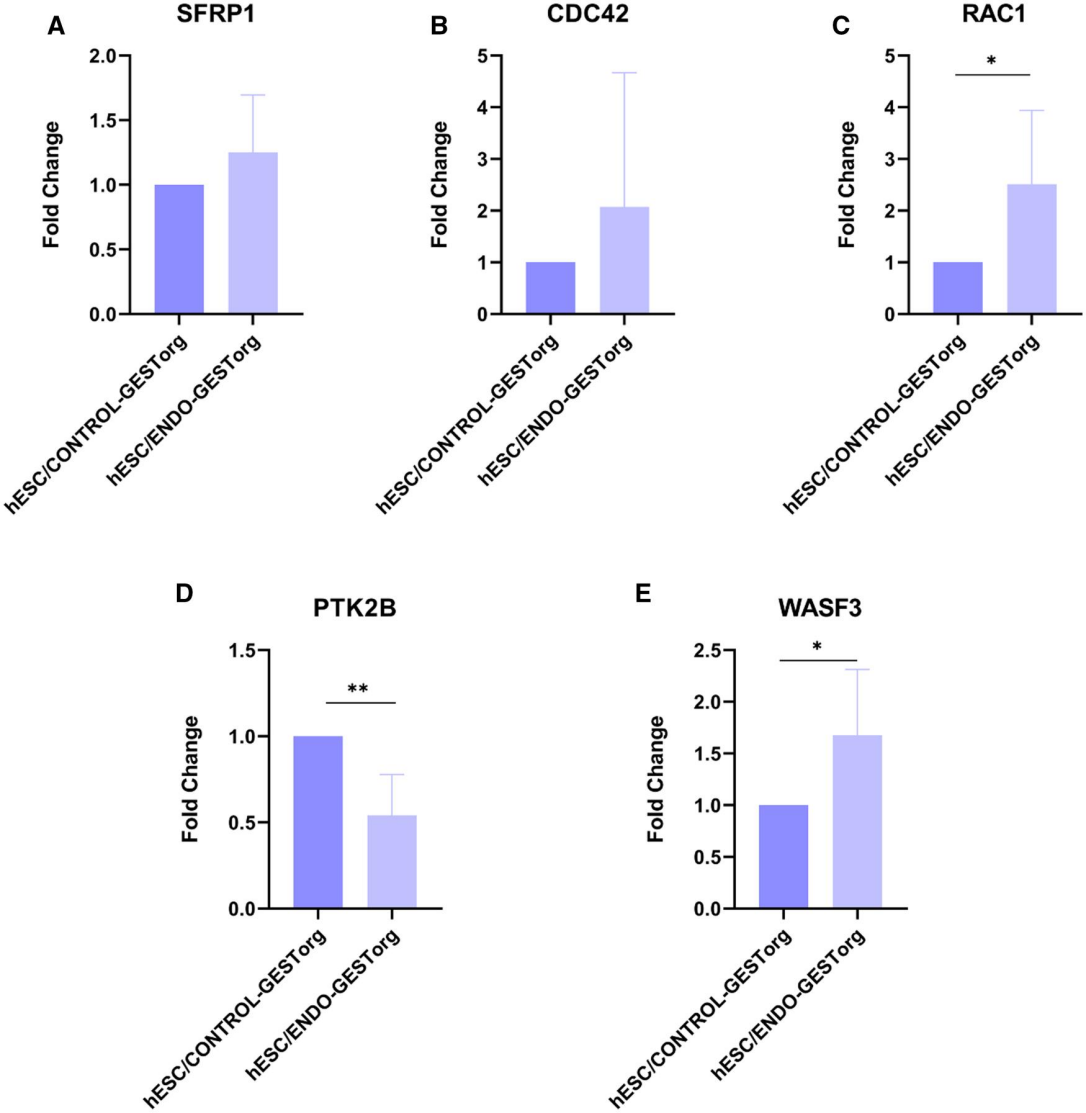
**Validation of micro (mi) RNA target genes in human endometrial stromal cells co-cultured with organoids.** Gene expression of (**A**) *SFRP1*, (**B**) *CDC42*, (**C**) *RAC1*, (**D**) *PTK2B*, and (**E**) *WASF3* was validated in hESC co-cultured with ENDO-GESTorg and compared with that in hESC co-cultured CONTROL-GESTorg. by qRT-PCR (n = 6 per group). Relative gene expression levels were calculated using the 2^−ΔΔCt method, normalizing target gene expression to GAPDH as a housekeeping gene. Statistical analysis was performed using the Mann–Whitney *U*-test. Statistical analysis was performed using the Mann–Whitney *U*-test. *P < 0.05; **P < 0.005. hESC, human endometrial stromal cells; ENDO-GESTorg, Gestational endometriosis organoids; CONTROL-GESTorg, Gestational control organoids.

Regarding the validation performed in hESC cells co-cultured with ENDO-GESTorg versus CONTROL-GESTorg, *SFRP1* was upregulated (FC = 1.25), consistent with the downregulation of its regulatory miRNA, miR-1290, in EVs secreted by ENDO-GEST organoids (Fig. 7A). Additionally, both *CDC42* and *RAC1* were upregulated (FC = 2.07 and 2.51; *P* < 0.05), respectively. *CDC42* is jointly regulated by miR-3065-5p and miR-4516, whereas *RAC1* is regulated by miR-320d; their increased expression is consistent with the reduced levels of these regulatory miRNAs in ENDO-GEST-derived EVs (Fig. 7B and C). *PTK2B* was downregulated (FC =0.47; *P* < 0.01), consistent with the upregulation of its regulatory miRNA, miR-335-5p (Fig. 7D). Finally, *WASF3* was also upregulated (FC = 1.68; *P* < 0.05), in line with the concomitant downregulation of miR-3065-5p and miR-1246 (Fig. 7E).

**Figure 8.**
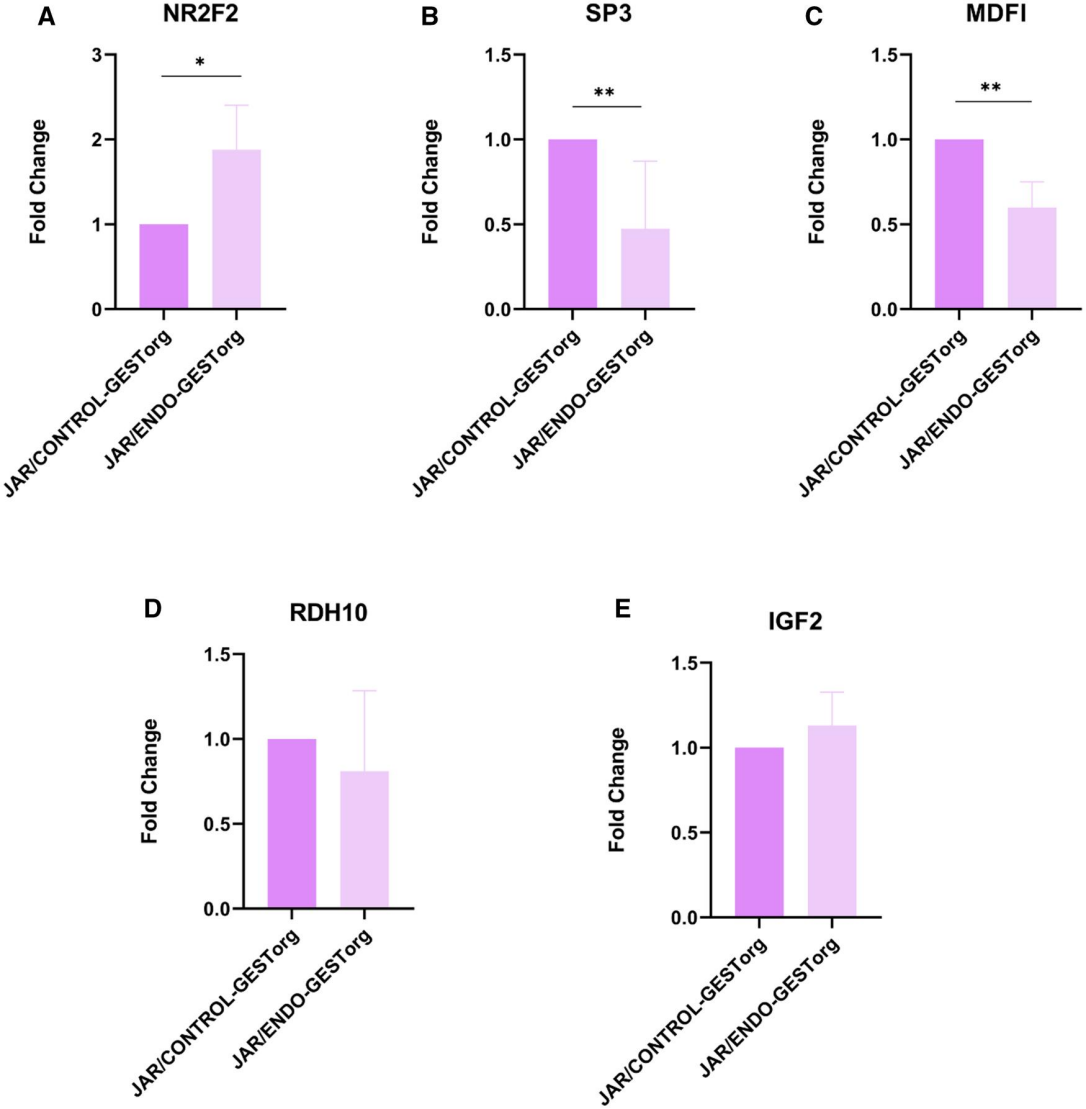
**Validation of micro (mi) RNA target genes in JAR choriocarcinoma cells co-cultured with organoids.** Gene expression of (**A**) *NR2F2*, (**B**) *SP3*, (**C**) *MDFI*, (**D**) *RDH10*, and (**E**) *IGF2* was validated in JAR co-cultured with ENDO-GESTorg and compared with that in JAR co-cultured CONTROL-GESTorg. by qRT-PCR (n = 6 per group). Relative gene expression levels were calculated using the 2^−ΔΔCt^ method, normalizing target gene expression to GAPDH as a housekeeping gene. Statistical analysis was performed using the Mann–Whitney *U*-test. *P < 0.05; **P < 0.005. JAR, JAR choriocarcinoma cells; ENDO-GESTorg, Gestational endometriosis organoids; CONTROL-GESTorg, Gestational control organoids.

Finally, in JAR cells co-cultured with ENDO-GESTorg versus CONTROL-GESTorg, *NR2F2* was upregulated (FC = 1.83; *P* < 0.05), in agreement with the downregulation of its regulatory miRNAs, miR-3065-5p and miR-1290 (Fig. 8A). In contrast, *SP3* was downregulated (FC = 0.47; *P* < 0.01), consistent with the upregulation of its regulatory miRNA, miR-191-5p (Fig. 8B). Similarly, *MDFI* and *RDH10* were also downregulated (FC = 0.6; *P* < 0.01 and 0.81, respectively), in line with their regulation by miR-335-5p and miR-191-5p in that order (Fig. 8C and D). Finally, *IGF2* was upregulated (FC = 1.31), consistent with the decreased levels of its regulatory miRNA, miR-320d (Fig. 8E).

## Discussion

Endometriosis is recognized not only as a cause of infertility but also as a contributor to adverse pregnancy outcomes (Vercellini *et al.*, 2023). Successful pregnancy requires appropriate endometrial–embryo crosstalk and a proper dialogue between all cell types comprising the local endometrial microenvironment; EVs have been identified as key mediators in this communication (Beal *et al.*, 2023). Thus, to elucidate the molecular mechanisms that could be linked to impaired implantation and placentation, we investigated the miRNA cargo of EVs secreted by our *in vitro* organoid model made with cells from patients with endometriosis. We found that the miRNA content of EVs secreted by the eutopic endometrium of patients with endometriosis was altered, which could underlie the frequent adverse pregnancy outcomes observed in these patients.

First, we verified that endometrial organoids secrete EVs into the culture medium. NTA revealed the presence of nanoparticles in the ENDO-GESTorg and Control-GESTorg conditioned media, with sizes 100–400 nm, which conforms to the standards of the International Society for Extracellular Vesicles (Théry *et al.*, 2018). Moreover, protein characterization revealed the presence of the transmembrane markers CD63, CD81, and CD9 in the nanoparticles obtained from ENDO-GESTorg and CONTROL-GESTorg conditioned culture media. These protein markers were absent in the nanoparticles isolated from non-conditioned media and Matrigel-only conditioned media, suggesting that EVs were exclusively secreted by the endometrial organoids. Finally, we confirmed the distinctive cup-shaped morphology of these EVs.miRNA-sequencing analysis revealed that miRNA content of ENDO-GESTevs exhibited a distinct transcriptomic profile compared to CONTROL-GESTevs. This analysis identified 289 DEmiRNAs in EVs secreted by ENDO-GESTorg. Among these, eight miRNAs were significantly different: six were downregulated (miR-1290, miR-1246, miR-320d, miR-4516, miR-12136, and miR-3065-5p), and two were upregulated (miR-191-5p and miR-335-5p) (Table 1). These miRNAs could be suggested as potential miRNA biomarkers for diagnosing endometriosis-related pregnancy complications.

**Table 1.** Key microRNAs in extracellular vesicles secreted by gestational eutopic endometrium of women with endometriosis and their potential role in pregnancy outcomes.

Name	Fold change	FDR *P*-value	Biological role	Impact on pregnancy outcomes
miR-1290	−3.726	1.25E−05	Promote EMT in endometrial epithelial cells and is also involved in modulation inflammation and promoting angiogenesis.	Its downregulation may play a role in impairing successful implantation.
miR-4516	−2.330	0.013	Pro-oncogenic properties.	Its downregulation may be associated with alterations in embryo totipotency or with disruptions in the inflammatory processes necessary for implantation.
miR-12136	−2.411	0.039	Pro-oncogenic properties.	Its downregulation may be associated with alterations in embryo totipotency or with disruptions in the inflammatory processes necessary for implantation.
miR-1246	−2.961	2.43E−05	Typically upregulated in syncytiotrophoblast to support proper differentiation.	Its downregulation could be involved in impairing placental development.
miR-320d	−2.329	6.27E−03	Promote proliferation and migration of endothelial cells in cancer.	Its downregulation could be linked to reduced angiogenic potential.
miR-3065-5p	−1.664	0.039	It is downregulated in placentas from SGA infants compared to normal placenta in women with preterm labour, as well as in placenta and trophoblast cells under inflammatory conditions.	Its downregulation could be involved in key processes in placental development and inflammatory conditions.
miR-191-5p	1.287	0.033	Anti-angiogenic effects on endothelial cells by inhibiting proliferation, migration, and tube formation.	Its upregulation might be associated with an increase in cell proliferation and migration, leading to pregnancy-related disorders.
miR-335-5p	1.551	0.033	Overexpression inhibits EMT in trophoblast cells and increases E-cadherin levels, which are critical for embryo implantation.	Its upregulation could be related to impaired pregnancy establishment in patients with endometriosis

EMT, epithelial to mesenchymal transition; SGA, small for gestational age.

Among the miRNAs downregulated in ENDO-GESTevs, miR-1290 may play a role in impairing successful implantation as it is known to promote EMT in endometrial epithelial cells and is also involved in modulating inflammation and promoting angiogenesis (Shi *et al.*, 2021). In turn, miR-4516 and miR-12136 have pro-oncogenic properties (Hao *et al.*, 2020; Li *et al.*, 2020) and therefore, their downregulation may be associated with alterations in embryo totipotency or with disruptions in the inflammatory processes necessary for implantation (Zhang *et al.*, 2021; Moore *et al.*, 2024). Furthermore, the downregulation of miR-1246 could be involved in impairing placental development given that this miRNA is typically upregulated in syncytiotrophoblast to support proper differentiation (Bhardwaj and Srivastava, 2024). In addition, exosomal miR-320d has been shown to promote proliferation and migration of endothelial cells in cancer; thus, its downregulation could be linked to reduced angiogenic potential (Wu *et al.*, 2024). Similarly, there are reports that miR-3065-5p is downregulated in placentas from SGA infants compared to those with normal placenta in women with preterm labour, as well as in placenta and trophoblast cells under inflammatory conditions (Östling *et al.*, 2019), suggesting that it could be involved in key processes in placental development.

Regarding the miRNAs upregulated in ENDO-GESTevs, miR-191-5p has anti-angiogenic effects on endothelial cells by inhibiting proliferation, migration, and tube formation (Gu *et al.*, 2017; Wu *et al.*, 2021), suggesting that its upregulation might be associated with pregnancy-related disorders. Finally, miR-335-5p overexpression has been shown to inhibit EMT in trophoblast cells and to increase E-cadherin levels, both of which are critical for embryo implantation (Tao *et al.*, 2025), suggesting that its upregulation in ENDO-GESTevs could be related to impaired pregnancy establishment in patients with endometriosis.

Prediction of target genes for these 8 significantly altered DEmiRNAs highlighted 3964 genes, and after functional enrichment, 789 biological processes significantly enriched in ENDO-GESevs were identified, many of which fell into four functional groups: endometrium–embryo communication, EMT–TEM, angiogenesis, and the immune system.

Regarding the first functional group, we noted that the most represented genes in endometrium–embryo communication are targeted by upregulated miRNAs in the ENDO-GESTevs, so if they are indeed suppressed in endometriosis, this could potentially affect pregnancy establishment. Firstly, *LIF* is strongly expressed in first-trimester decidua and trophoblast and mediates the interaction between them (Aikawa *et al.*, 2024). *SP3* regulates *HSD17B2* expression in endometrial epithelial cells, establishing a low-oestrogenic environment that supports implantation (Cheng *et al.*, 2006). *RDH10* is crucial for endometrial function because it promotes decidual transformation and stromal cell proliferation and moreover, its increased expression during implantation may modulate immune tolerance (Nakajima *et al.*, 2016). Among the genes targeted by downregulated miRNAs, *IGF2* enhances proliferation, migration, and invasion in endometriotic cells and is upregulated in foetal tissues obtained from cases of miscarriage (Vasconcelos *et al.*, 2019). Finally, *NR2F2* must be tightly regulated given its substantial involvement in embryogenesis and organogenesis (Polvani *et al.*, 2019).

During implantation, the endometrial luminal epithelium undergoes EMT, enabling cells to acquire migratory and invasive properties, and allowing the blastocyst to penetrate the epithelial barrier and invade the endometrial stroma (Oghbaei *et al.*, 2022). Similarly, trophectoderm cells undergo the EMT to acquire an invasive phenotype and anchor the placenta (Li *et al.*, 2020). In parallel, decidualized stromal cells adopt a more mesenchymal and motile phenotype to support implantation (Owusu-Akyaw *et al.*, 2019). The most represented genes targeted by upregulated miRNAs in the EMT–TEM functional group included *PTK2B*, which regulates migration and invasion in cancer cells (Gil-Henn *et al.*, 2024), and *F11R*, which regulates endothelial and epithelial tight junctions, leukocyte migration, and angiogenesis. The suppression of these genes has been shown to reduce EMT and, consequently, metastasis in patients with endometriosis (Czubak-Prowizor *et al.*, 2022).

Within the group of genes targeted by downregulated miRNAs: increased *SFRP1* expression has been observed in placentas with intrauterine growth restriction (Lan *et al.*, 2024); *WASF3* is involved in mitochondrial respiration (Wang *et al.*, 2023); and excessive *RAC1* contributes to endometrial cell stiffness in cases of preeclampsia (Raja Xavier *et al.*, 2024) and, together with *CDC42*, is activated by *PLAC8*. The expression of the latter has been found to be upregulated in trophoblast cells obtained from placentas from women with preeclampsia, as was the expression of both these genes in the same tissues (Chang *et al.*, 2018).

Angiogenesis is essential for embryo implantation and early placental development, ensuring proper oxygen and nutrient supply to support pregnancy (Kim *et al.*, 2013; Chen *et al.*, 2023). Among the genes targeted by upregulated miRNAs in the angiogenesis functional group, *VEGFA* is one of the most highly represented. *VEGFA* is secreted by decidual stromal cells after implantation, promoting endothelial proliferation and vascular network formation. Downregulation of *VEGFA* has been associated with recurrent implantation failure (RIF) and recurrent miscarriage (Guo *et al.*, 2021). Inhibition of *NOS3* has also been shown to induce preeclampsia phenotypes in pregnant rat models (Ssengonzi *et al.*, 2024), while *CDH5* upregulation in cytotrophoblasts is essential for the remodelling uterine arteries (Varshavsky *et al.*, 2020) and its downregulation could affect placenta development. In addition, among the genes targeted by both downregulated and upregulated miRNAs, *NPR1* is crucial for trophoblast development and its expression is downregulated in placentas from preeclamptic patients (Yang *et al.*, 2021). Similarly, knocking out *KDR* in experimental models results in defective placental vasculature and consequent embryonic lethality (Keshavarz and Yavarian, 2019; Bhardwaj and Srivastava, 2024).

A tolerogenic immune environment in the female reproductive tract during pregnancy is essential to suppress inflammation and protect embryo and foetal survival (Moffett and Shreeve, 2023). Among the most represented genes targeted by upregulated miRNAs in the Immune System functional group is *IL4*, which is essential for pregnancy maintenance by supporting trophoblast repair, proliferation, and survival (Piccinni *et al.*, 2021). *LGALS9* modulates immune tolerance and its decreased levels during early gestation are associated with miscarriage and disrupted Th1/Th2 balance (Heusschen *et al.*, 2013; Mendoza *et al.*, 2024)*. IL6* promotes trophoblast invasion, vascular remodelling, and immune–endocrine crosstalk (Vilotić *et al.*, 2022). Among genes targeted by downregulated miRNAs, *HMGB1* is highly expressed in the decidua of women with recurrent pregnancy loss (Zhu *et al.*, 2021) and its expression is elevated in placentas from women who had severe preeclampsia (Shirasuna *et al.*, 2020). In turn, *HLA* class Ib molecules, such as *HLA-G, HLA-E*, and *HLA-F*, play critical roles in establishing maternal–foetal immune tolerance and supporting pregnancy progression (Yang *et al.*, 2022).

In summary, the KEGG pathway enrichment analysis of target genes from DEmiRNAs in ENDO-GESTevs revealed significant enrichment of signalling pathways previously associated with essential reproductive and cellular functions. Notably, pathways such as Wnt, oestrogen, and PI3K–Akt signalling, key regulators of implantation, decidualization, and endometrial remodelling, were strongly enriched in our experimental group. Additionally, several pathways related to immune modulation (e.g. IL-17 and chemokine signalling), cell adhesion, hormone signalling, and stem cell pluripotency were also significantly represented, highlighting the possible involvement of these DEmiRNAs with key processes of endometrial receptivity and maternal–foetal communication. These findings suggest that targeted therapeutic strategies could be aimed at restoring endometrial communication and improving reproductive outcomes in endometriosis.

The experiments performed with co-cultures appear to support the hypothesis that the content of EVs produced by the endometrium can modulate gene expression in other cell types and in the embryo, and that this regulation may be altered in pathological conditions such as endometriosis. However, it is important to note, that this study has several limitations. The use of endometrial organoids represents an *in vitro* model that cannot fully replicate the complex intrauterine environment. EVs were pooled from different patients, which does not consider individual biological variability, but allows the assessment of variability between pools and study groups. However, pooling in transcriptomic studies limits statistical interpretation, and a larger sample size would be desirable in a future study to further increase the robustness of the findings. In addition, in light of the encouraging functional results, it would be desirable to incorporate additional cell types to further improve the model, as well as to perform additional assays involving functional manipulation to enhance mechanistic depth. Despite these limitations, the findings provide a solid foundation for exploring, for the first time, an under-investigated area and may lay the groundwork for the development of targeted therapeutic strategies aimed at restoring endometrial communication and improving reproductive outcomes in affected women. Furthermore, the altered miRNA cargo identified in endometrial EVs could be evaluated as potential non-invasive biomarkers in patient-derived samples, such as serum or plasma, to diagnose endometriosis and related pregnancy complications.

Based on these results, we suggest that the miRNA content of EVs secreted by organoids derived from eutopic endometrium from women with endometriosis may be altered. These potential alterations could play a role in the pathogenesis of endometriosis and might influence key processes required for a successful pregnancy establishment and maintenance; however, further studies are needed to confirm these effects.

## Supplementary Material

hoag040_Supplementary_Data

## Data Availability

The data underlying this article are available in Gene Expression Omnibus (GEO) at https://www.ncbi.nlm.nih.gov/geo/query/acc.cgi?&acc=GSE302961, under accession number GSE302961.
