# Supplementary material for: Altered miRNA cargo of endometrial extracellular vesicles in patients with endometriosis: potential implications for pregnancy outcomes
Source: Hum Reprod Open. 2026 May 7;2026(3):hoag040. doi: 10.1093/hropen/hoag040 (PMC13249616; doi:10.1093/hropen/hoag040)
Supplement: hoag040_Supplementary_Data [file hoag040_supplementary_data.zip › HRO-25-0373-R2-SuppTableS1.docx]

**Supplementary Table S1. The basic demographic parameters of the patients.**

| PATIENT | AGE | BMI | PATHOLOGY | YEARS OF INFERTILITY | ENDOMETRIAL THICKNESS | PARITY | POOL |
| --- | --- | --- | --- | --- | --- | --- | --- |
| ENDO 1 | 32 | 21.2 | Endometriosis stage III/IV | 6 months | - | G0 | ENDO-GESTevs-1 |
| ENDO 2 | 38 | 30.30 | Endometrioma | 6 months | 14 mm | G1A1 | ENDO-GESTevs-2 |
| ENDO 3 | 33 | 21.09 | Endometrioma/deep endometriosis | 2 years | 8mm | G0 | ENDO-GESTevs-3 |
| ENDO 4 | 36 | 22.23 | Endometrioma | 4 years | 11 mm | G0 | ENDO-GESTevs-1 |
| ENDO 5 | 33 | 19.45 | Endometriosis stage III/IV | 6 months | - | G0 | ENDO-GESTevs-1 |
| ENDO 6 | 32 | 17.80 | Endometriosis stage III/IV | 2.5 years | 8mm | G0 | ENDO-GESTevs-2 |
| ENDO 7 | 40 | 20.86 | Endometriosis stage III/IV | 2.5 years | 9.6 mm | G0 | ENDO-GESTevs-3 |
| ENDO 8 | 30 | 18.1 | Endometrioma | 6 months | - |  | ENDO-GESTevs-4 |
| ENDO 9 | 39 | 22.80 | Endometriosis stage III/IV | 2.5 years | 7.6 mm | G0 | ENDO-GESTevs-2 |
| ENDO 10 | 30 | 22.80 | Endometriosis stage III/IV | 1 year | 8 mm | GO | ENDO-GESTevs-4 |
| ENDO 11 | 40 | 19.60 | Endometriosis stage III/IV | 1 year | 10 mm | G0 | ENDO-GESTevs-4 |
| ENDO 12 | 38 | 20.80 | Endometriosis stage III/IV | 3 years | 6 mm | G0 | ENDO-GESTevs-2 |
| ENDO 13 | 39 | 27.39 | Endometrioma | 6 months | 10.4 mm | G0 | ENDO-GESTevs-1 |
| ENDO 14 | 36 | - | Endometriosis stage III/IV | 1 year | - | G0 | ENDO-GESTevs-4 |
| ENDO 15 | 34 | - | Endometriosis stage III/IV | 2 years | 12mm | G0 | ENDO-GESTevs-3 |
| ENDO 16 | 31 | 23.4 | Endometrioma | 2 years | 9 mm | G0 | ENDO-GESTevs-3 |
| PATIENT | **AGE** | **BMI** | **PATHOLOGY** | **YEARS OF INFERTILITY** | **ENDOMETRIAL THICKNESS** | **PARITY** | **POOL** |
| CONTROL 1 | 31 | 29.49 | Healthy oocyte donor | N/A | - | G2P1A0EA1 | CONTROL-GESTevs-1 |
| CONTROL 2 | 19 | 26.44 | Healthy oocyte donor | N/A | - | G1P1A0EA0 | CONTROL-GESTevs-1 |
| CONTROL 3 | 24 | 20.83 | Healthy oocyte donor | N/A | - | G1P1A0EA1 | CONTROL-GESTevs-2 |
| CONTROL 4 | 21 | 20.57 | Healthy oocyte donor | N/A | - | G1P0A0EA1 | CONTROL-GESTevs-1 |
| CONTROL 5 | 29 | 22.33 | Healthy oocyte donor | N/A | - | G1P0A0EA0 | CONTROL-GESTevs-3 |
| CONTROL 6 | 24 | 18.29 | Healthy oocyte donor | N/A | - | G2P1A0EA1 | CONTROL-GESTevs-2 |
| CONTROL 7 | 34 | 22.5 | Healthy oocyte donor | N/A | - | G2P1A0EA1 | CONTROL-GESTevs-3 |
| CONTROL 8 | 27 | 28.52 | Healthy oocyte donor | N/A | - | G2P1A0EA1 | CONTROL-GESTevs-2 |
| CONTROL 9 | 32 | 22.83 | Healthy oocyte donor | N/A | - | - | CO242NTROL-GESTevs-2 |
| CONTROL 10 | 22 | 22.61 | Healthy oocyte donor | N/A | - | G1P1A0EA0 | CONTROL-GESTevs-3 |
| CONTROL 11 | 29 | 22.52 | Healthy oocyte donor | N/A | - | G1P1A0EA0 | CONTROL-GESTevs-1 |
| CONTROL 12 | 24 | 22.13 | Healthy oocyte donor | N/A | - | - | CONTROL-GESTevs-1 |
| CONTROL 13 | 21 | 24.20 | Healthy oocyte donor | N/A | - | - | CONTROL-GESTe21vs-3 |
| CONTROL 14 | 24 | 19.81 | Healthy oocyte donor | N/A | - | G1P1A0EA1 | CONTROL-GESTevs-2 |
| CONTROL 15 | 29 | - | Healthy oocyte donor | N/A | - | G3P1A0EA2 | CONTROL-GESTevs-3 |

NA: Not applicable; G: Gestation; P: Parity; A: miscarriage; EA: elected abortion
