# Supplementary material for: Altered miRNA cargo of endometrial extracellular vesicles in patients with endometriosis: potential implications for pregnancy outcomes
Source: Hum Reprod Open. 2026 May 7;2026(3):hoag040. doi: 10.1093/hropen/hoag040 (PMC13249616; doi:10.1093/hropen/hoag040)
Supplement: hoag040_Supplementary_Data [file hoag040_supplementary_data.zip › Supplementary Figure S1.pdf]

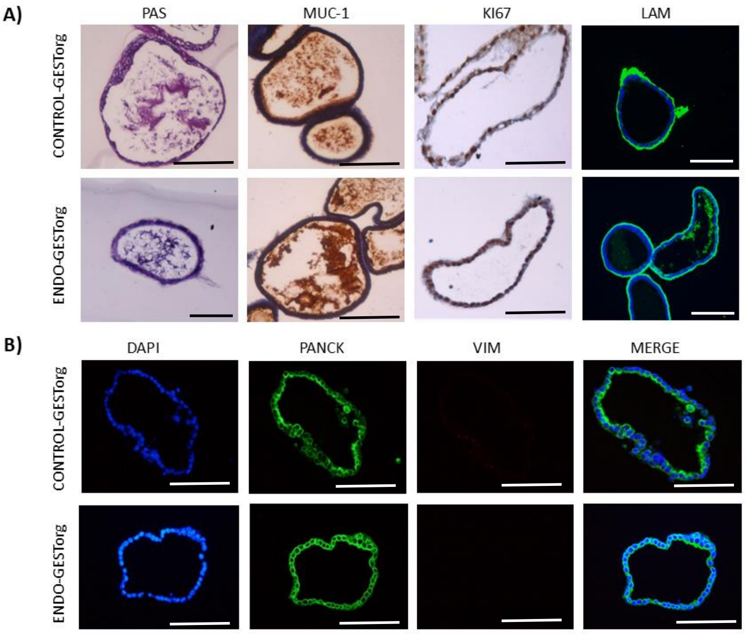

Supplementary Figure S1. Characterisation of gestational endometrial organoids derived from patients with endometriosis versus healthy controls. Representative images of (A) Periodic acid–Schiff staining, and MUC-1, Ki67, and laminin expression by immunohistochemistry. (B) pan-cytokeratin and vimentin staining by immunofluorescence in the ENDO-GESTorg and CONTROL-GESTorg groups. Organoids accurately replicate the morphological and functional characteristics of endometrial epithelial tissue, both CONTROL-GESTorg and ENDO-GESTorg produce periodic acid-Schiff (PAS) material and mucin, retain their proliferative capacity (Ki-67), maintain apicobasal polarity (laminin), and express only epithelial markers (pan-cytokeratin) but not stromal markers (vimentin). The scale bars represent 100 µm. Liver, endometrium, and breast cancer samples were used as positive controls.; CONTROL-GESTorg: Gestational control organoids.
