# Supplementary material for: Altered miRNA cargo of endometrial extracellular vesicles in patients with endometriosis: potential implications for pregnancy outcomes
Source: Hum Reprod Open. 2026 May 7;2026(3):hoag040. doi: 10.1093/hropen/hoag040 (PMC13249616; doi:10.1093/hropen/hoag040)
Supplement: hoag040_Supplementary_Data [file hoag040_supplementary_data.zip › Supplementary Figure S2.pdf]

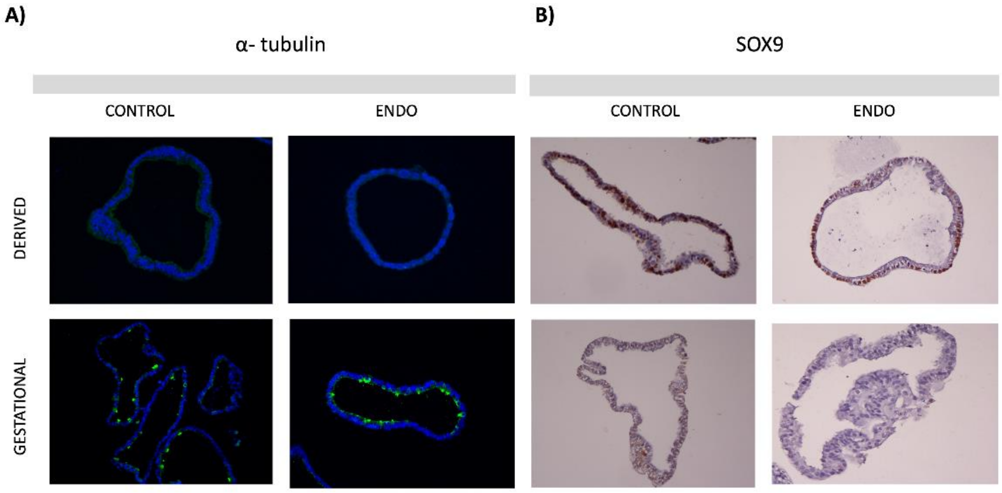

Supplementary Figure S2. Characterisation of gestational endometrial organoids derived from women with endometriosis and healthy controls. Representative images of (A) SOX 9 and (B) α-tubulin protein expression in ENDO-GESTorg and CONTROL-GESTorg. α-tubulin expression is expected to be increased in organoids differentiated toward the gestational phase compared to those merely derived, while SOX9 expression should be reduced.The scale bars represent 100 µm. Liver, endometrium, and breast cancer samples were used as positive controls. CONTROL: Control organoids; ENDO: Endometriosis organoids; DERIVED: Organoids non differentiated to the gestational phase; GESTATIONAL: Organoids differentiated to the gestational phase.
