# Supplementary material for: Altered miRNA cargo of endometrial extracellular vesicles in patients with endometriosis: potential implications for pregnancy outcomes
Source: Hum Reprod Open. 2026 May 7;2026(3):hoag040. doi: 10.1093/hropen/hoag040 (PMC13249616; doi:10.1093/hropen/hoag040)
Supplement: hoag040_Supplementary_Data [file hoag040_supplementary_data.zip › Supplementary Figure S4.pdf]

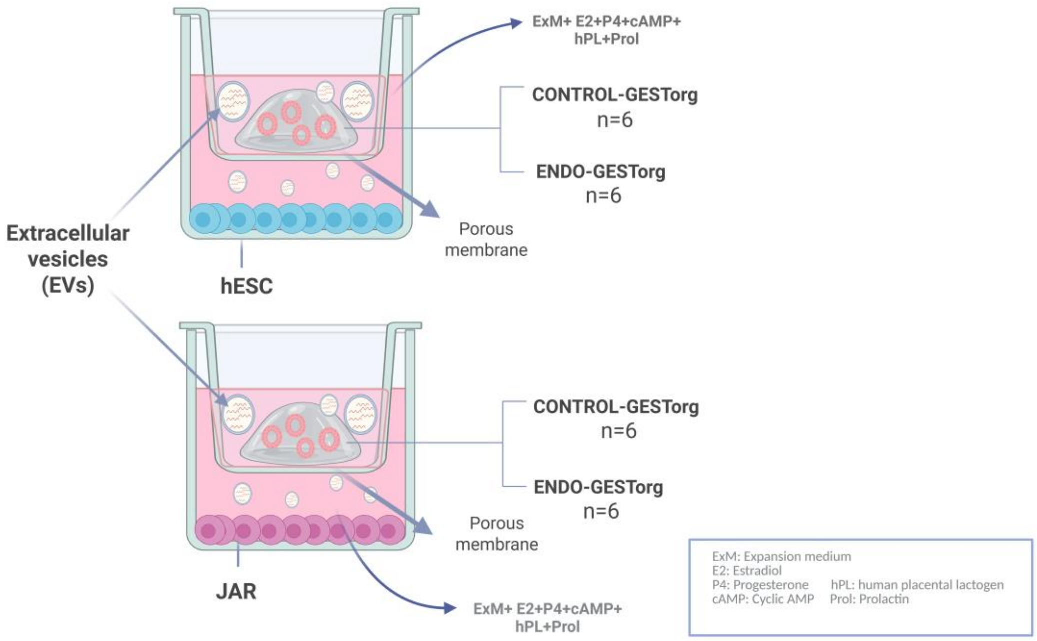

Supplementary Figure S4. Schematic representation of the experimental design for co-culture models involving endometrial epithelial organoids, hESCs, and JAR cells.
