# Supplementary material for: Altered miRNA cargo of endometrial extracellular vesicles in patients with endometriosis: potential implications for pregnancy outcomes
Source: Hum Reprod Open. 2026 May 7;2026(3):hoag040. doi: 10.1093/hropen/hoag040 (PMC13249616; doi:10.1093/hropen/hoag040)
Supplement: hoag040_Supplementary_Data [file hoag040_supplementary_data.zip › Supplementary Table S2.docx]

**Supplementary Table S2. Primer description.**

| Gene |  | Primer sequence | Commercial reference |
| --- | --- | --- | --- |
| *IGF2* | Forward | CTGGAGACGTACTGTGCTA | Invitrogen, Carlsbad, CA, USA |
|  | Reverse | CCAGGTGTCATATTGGAAGAAC |  |
| *nr2f2* | Forward | CAAGGCCATAGTCCTGTTC | Invitrogen, Carlsbad, CA, USA |
|  | Reverse | CTTCCAAAGCACACTGAGA |  |
| *ptk2b* | Forward | AGAGAGCTGCAGCATAGA | Invitrogen, Carlsbad, CA, USA |
|  | Reverse | CGATTCAGGACCACATCTTC |  |
| *wasf3* | Forward | CAACAGGTTGTCTCAGAGTG | Invitrogen, Carlsbad, CA, USA |
|  | Reverse | GAGTAGCCGGGTAAGAGTAA |  |
| *mdfi* | Forward | AGTAAGAGCAGCAGCAAATC | Invitrogen, Carlsbad, CA, USA |
|  | Reverse | TCAGGAACTCGCAGAACA |  |
| *rdh10* | Forward | TCTACCTGACGGCTGAAA | Invitrogen, Carlsbad, CA, USA |
|  | Reverse | GGCACAGTAATCCTCAACTC |  |
| *sfrp1* | Forward | CGATGGTGCTGTTGGTAAA | Invitrogen, Carlsbad, CA, USA |
|  | Reverse | AGCAGTCTCTGGAGTGATAG |  |
| *cdc42* | Forward | CGATGGTGCTGTTGGTAAA | Invitrogen, Carlsbad, CA, USA |
|  | Reverse | AGCAGTCTCTGGAGTGATAG |  |
| *rac1* | Forward | GAGACGGAGCTGTAGGTAAA | Invitrogen, Carlsbad, CA, USA |
|  | Reverse | CATCTGTTTGCGGATAGGATAG |  |
| *LIF* | Forward | GTACTGGCACTCGTTCGATC | Invitrogen, Carlsbad, CA, USA |
|  | Reverse | GAGTCTTTCTCCTCCCTGTCC |  |
| *sp3* | Forward | GTAAAGAAGGTGGTGGAAGAG | Invitrogen, Carlsbad, CA, USA |
|  | Reverse | ATGTGAGGTCTTCCCATAGA |  |
| *rac1* | Forward | GAGAGCGAGCTGTAGGTAAA | Invitrogen, Carlsbad, CA, USA |
|  | Reverse | CATCTGTTTGCGGATAGGATAG |  |
| *gapdh* | Forward | AGATCAAGAAGGTGGTGAAG | Invitrogen, Carlsbad, CA, USA |
|  | Reverse | TTGTCATACCAGGAAATGAGC |  |
